# Establishing a method of short-term rainfall forecasting based on GNSS-derived PWV and its application

**DOI:** 10.1038/s41598-017-12593-z

**Published:** 2017-09-29

**Authors:** Yibin Yao, Lulu Shan, Qingzhi Zhao

**Affiliations:** 1 School of Geodesy and Geomatics, Wuhan University, 129 Luoyu Road, Wuhan, 430079 China; 2 Key Laboratory of Geospace Environment and Geodesy, Ministry of Education, Wuhan University, 129 Luoyu Road, Wuhan, 430079 China; 3 College of Geomatics, Xi’an University of Science and Technology, 58 Yanta Road, Xi’an, 710054 China

## Abstract

Global Navigation Satellite System (GNSS) can effectively retrieve precipitable water vapor (PWV) with high precision and high-temporal resolution. GNSS-derived PWV can be used to reflect water vapor variation in the process of strong convection weather. By studying the relationship between time-varying PWV and rainfall, it can be found that PWV contents increase sharply before raining. Therefore, a short-term rainfall forecasting method is proposed based on GNSS-derived PWV. Then the method is validated using hourly GNSS-PWV data from Zhejiang Continuously Operating Reference Station (CORS) network of the period 1 September 2014 to 31 August 2015 and its corresponding hourly rainfall information. The results show that the forecasted correct rate can reach about 80%, while the false alarm rate is about 66%. Compared with results of the previous studies, the correct rate is improved by about 7%, and the false alarm rate is comparable. The method is also applied to other three actual rainfall events of different regions, different durations, and different types. The results show that the method has good applicability and high accuracy, which can be used for rainfall forecasting, and in the future study, it can be assimilated with traditional weather forecasting techniques to improve the forecasted accuracy.

## Introduction

Water vapor is an important component of the atmosphere, which affects the radiation balance, energy transportation, and the formation of cloud and precipitation^1^. Water vapor has a very low content but a complex spatial distribution in atmosphere, and it plays an important role in atmospheric processes such as rain, adverse weather, and global climate variation^2,3^.Traditional atmosphere sensing techniques such as radiosonde and microwave radiometer (MWR) have shortcomings in reflecting the continuous transformations of atmospheric water vapor because of their low spatiotemporal resolution^4^. Global navigation satellite system (GNSS) has several advantageous  characteristics (*i.e*., all weather applications, high accuracy, and low cost), and it has been recognized as an efficient approach to estimate precipitable water vaper (PWV)^5–8^.

The GNSS-derived PWV can reflect the inflow and outflow of water vapor in a vertical air column above a certain area^9,10^, which is important in studying severe water vapor variations^11^. GNSS-derived PWV have been widely used in meteorology applications such as data assimilation and numerical model calibration efforts, climate studies, and precipitation forecasts and analysis. Different types of rainfall process, especially extreme rainfall events have different great impacts on all aspects of living and property^12^. It brings serious destruction and enormous economic loses^13^.Thus, in this paper, we study the relationship between GNSS-derived PWV and rainfall, and establish a short-term rainfall forecasting method. The specific aim is to forecast rainfalls in advance and warn the severe convection weather timely. In the further research, the method can be assimilated with the traditional weather forecasting techniques such as Numerical Weather Prediction (NWP) to improve the forecasted accuracy.

Several studies have been done to analyze the potential of GNSS-derived PWV to forecast rainfalls. Supart *et al*.^14^ showed GNSS-PWV has a relationship with the measured rainfall. Wang *et al*.^3,15^ concluded that rapid rise in the GPS-PWV in the long term low-level data predicted the arrival of rainfall and was therefore useful in weather forecasts. Cao *et al*.^16^ found that GPS-PWV could be used to improve the near real-time forecast/short term forecast of rainfalls. Shi *et al*.^8^ used an empirical real-time GPS PPP-derived PWV threshold for rainfall forecasting. Shoji *et al*.^17^ and Kanda^18^ verified that, when PWV reached a certain threshold, the probability of rainfall would be quickly increased. Seco *et al*.^19^ used nine consecutive years of data in NE Spain to achieve short-term rainfall forecast by using GNSS-PWV data and surface pressure data. Liu *et al*.^20^ concluded that with levels of total atmospheric water vapor above 25 mm and an increase in water vapor higher than 5 mm, the probability of rainfall is about 50%. Benevides *et al*.^21^ used GNSS-PWV to forecast rainfalls, and the forecasted correct rate was about 75%. Although the relationship between PWV and rainfall is pointed out, rigorous studies are yet to be done in rainfalls forecasting using PWV data. Therefore, a new method of short-term rainfall forecasting based on GNSS-derived PWV is proposed here.

In this paper, a new method which has high accuracy in short-term rainfall forecasting is established and verified. The paper is organized as follows. After obtaining the hourly GNSS-derived PWV and rainfall data, the relationship between GNSS-PWV and rainfall is analyzed. Then, a method of short-term rainfall forecasting is proposed based on above analysis. After that, the method is tested using PWV and actual rainfall information in Zhejiang. Finally, the method is applied to three different rainfall events to verify its reliability and applicability, and the conclusion is drawn.

## Data acquisition

### PWV retrieval

The troposphere delay effect on GPS signals can be divided into a hydrostatic part and a wet part^22^ by

1 $${\rm{ZTD}}={\rm{ZHD}}+{\rm{ZWD}}$$

Where, ZTD is the zenith total delay, ZHD is the zenith hydrostatic delay, and ZWD is the zenith wet delay.

Usually, ZHD can be calculated with high accuracy based on Saastamoinen model^6^, which can be expressed as:

2 $${\rm{ZHD}}=\frac{0.0022768P}{1-0.00266\,\cos \,(2\phi )-0.00028H}$$

Where, P, ϕ, and H represent the station total pressure, station latitude, and station height, respectively.

PWV is related to ZWD via a conversion factor by:

3 $${\rm{PWV}}={\rm{\pi }}\cdot {\rm{ZWD}}$$

4 $${\rm{\pi }}=\frac{{10}^{6}}{{\rho }_{w}{R}_{v[({K}_{3}/{T}_{m})+{K}_{2}^{\text{'}}]}}$$

Where, ${\rm{\pi }}\,\,$is the conversion factor;$\,{\rho }_{w}\,\,$is the density constant of liquid water (103 ${\rm{kg}}/{m}^{3}$); ${R}_{v}$ is the gas constant for water vapor (461 ${\rm{J}}\cdot {{\rm{kg}}}^{-1}\cdot {{\rm{K}}}^{-1}$); ${K}_{2}^{\text{'}}\,\,$and *K*_3_ are the atmospheric refractivity constants^23^, where $\,{K}_{2}^{\text{'}}=16.48K\cdot hP{a}^{-1}$ and $\,{K}_{3}=(3.776\pm 0.014)\times {10}^{5}{K}^{2}hP{a}^{-1}$, respectively; and *T*_*m*_ is the weighted mean temperature of the troposphere.

$\,{T}_{m}\,\,$can be expressed as^6^:

5 $${T}_{m}=\frac{{\int }^{}(e/T)dz}{{\int }^{}(e/{T}^{2})dz}$$

Where, e is the water vapor pressure, T is the absolute temperature, and dz is the integral path.

Generally, it can be calculated by empirical equation from Bevis^6^ using the ground temperature *T*_*s*_ as follows^24,25^:

6 $${T}_{m}=a+b{T}_{s}$$

The equation is usually established under the constraints of sounding data and reanalysis data in the specific area. In this paper, the parameters a and b is determined with radiosonde data from the experimental area, and the following equation is obtained:

7 $${T}_{m}=44.05+0.81{T}_{s}$$

### Data introduction

Zhejiang Province is located in the subtropical monsoon climate zone. The air in Zhejiang is moist due to the abundant precipitation, and various meteorological disasters often occur. Zhejiang has amongst the highest annual average precipitation in China which is about 1600 mm, and most of the rainfalls are concentrated in May and June. The land area of Zhejiang is 10.55 million km^2^, which accounts for 1.1% of the land mass of China.

**Figure 1:**
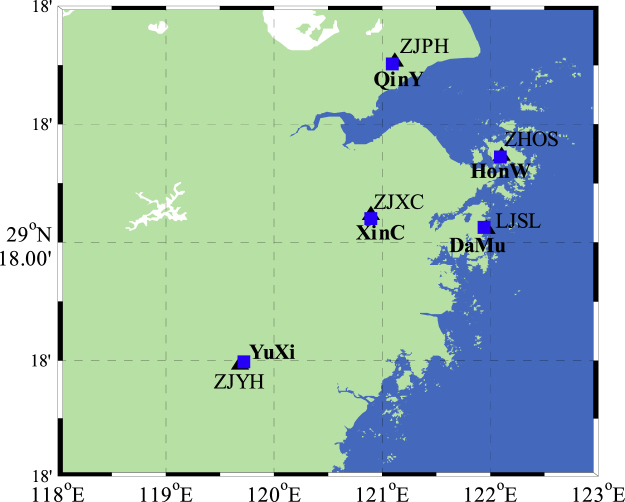
GNSS stations (black triangles) and nearby rainfall stations (blue squares) [the figure is plotted by MATLAB 2016a (https://cn.mathworks.com/products/matlab.html)].

Five stations (LJSL, ZHOS, ZJPH, ZJXC, and ZJYH) are selected in Zhejiang CORS network (http://www.zjcors.com/) with their nearby rainfall stations (Fig. 1). Table 1 lists the coordinate information of the stations. GNSS observations with a sampling interval of 30 s from 1 September 2014 to 31 August 2015 and hourly rainfall information from rainfall stations are selected for experiments. The GNSS observation data are processed by Precise Point Positioning (PPP) data processing software. By changing the time interval to one hour in data processing process and using equations in section 1.1, the hourly PWV sequence is obtained.

**Table 1:** Information for each station, including the longitude, latitude, and altitude.

Station	Longitude(°)	Latitude(°)	Altitude(m)
LJSL	121.9682	29.4198	74.02
ZHOS	122.1073	30.0354	53.02
ZJPH	121.1128	30.8254	17.52
ZJXC	120.8906	29.5319	170.10
ZJYH	119.6900	28.2660	130.08

## Analysis of the relationship between GNSS-PWV and rainfall

### Preliminary analysis of the relationship between GNSS-PWV and rainfall

**Figure 2:**
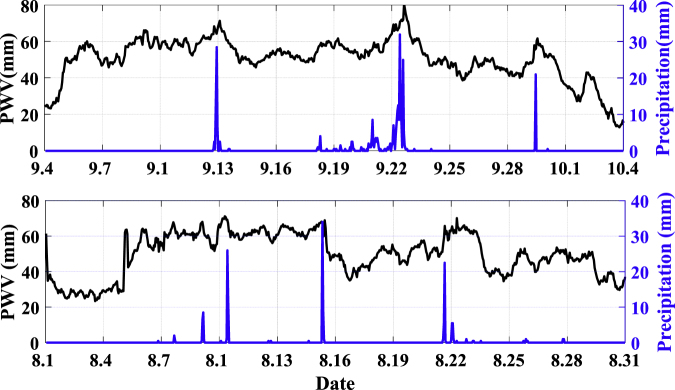
The relationship between GNSS-derived PWV and several rainfall events at station LJSL. Black line represents PWV values, and blue line represents hourly rainfall.

To explore the relationship between GNSS-derived PWV and rainfall, station LJSL is taken as an example to analyze the time-varying characteristic of PWV during several rainfall events (Fig. 2).

It can be seen from Fig. 2 that PWV usually rises significantly before a rainfall and decreases after the rainfall, and most of maximum rainfall occurs near the PWV peak. Taking the rainfall occurred in 13 September 2014 as an example to analyze the PWV variations in a rainfall process. Before the rainfall, PWV time series fluctuate below 60 mm during September 10 to September 12. At September 12, PWV falls to 50 mm, and then, it begins to increase rapidly till September 13 to the peak value of 70 mm. When the rainfall stops, PWV decreases and down to the minimum of 45 mm. Then the PWV time series fluctuate at about 50 mm until the next rainfall occur. Similar process of PWV variations can also be seen in other rainfalls.

PWV will increase when rainfall occurs, but not all high PWV values indicate the occurrence of rainfalls. For example, a sharp increment causes a high PWV level about 60 mm around August 5 in the second subgraph, but no rainfall is recorded during the period. The rise of water vapor is only one of the necessary prerequisites for the occurrence of rainfalls. Some external dynamic factors such as thermodynamic variation are also necessary to trigger rainfalls. If the condition of external dynamic factors are not satisfied, the rainfall events might not happen even when the GNSS-PWV values are at a high level^17^.

### Analysis of GNSS-PWV variation before several rainfall events

**Figure 3:**
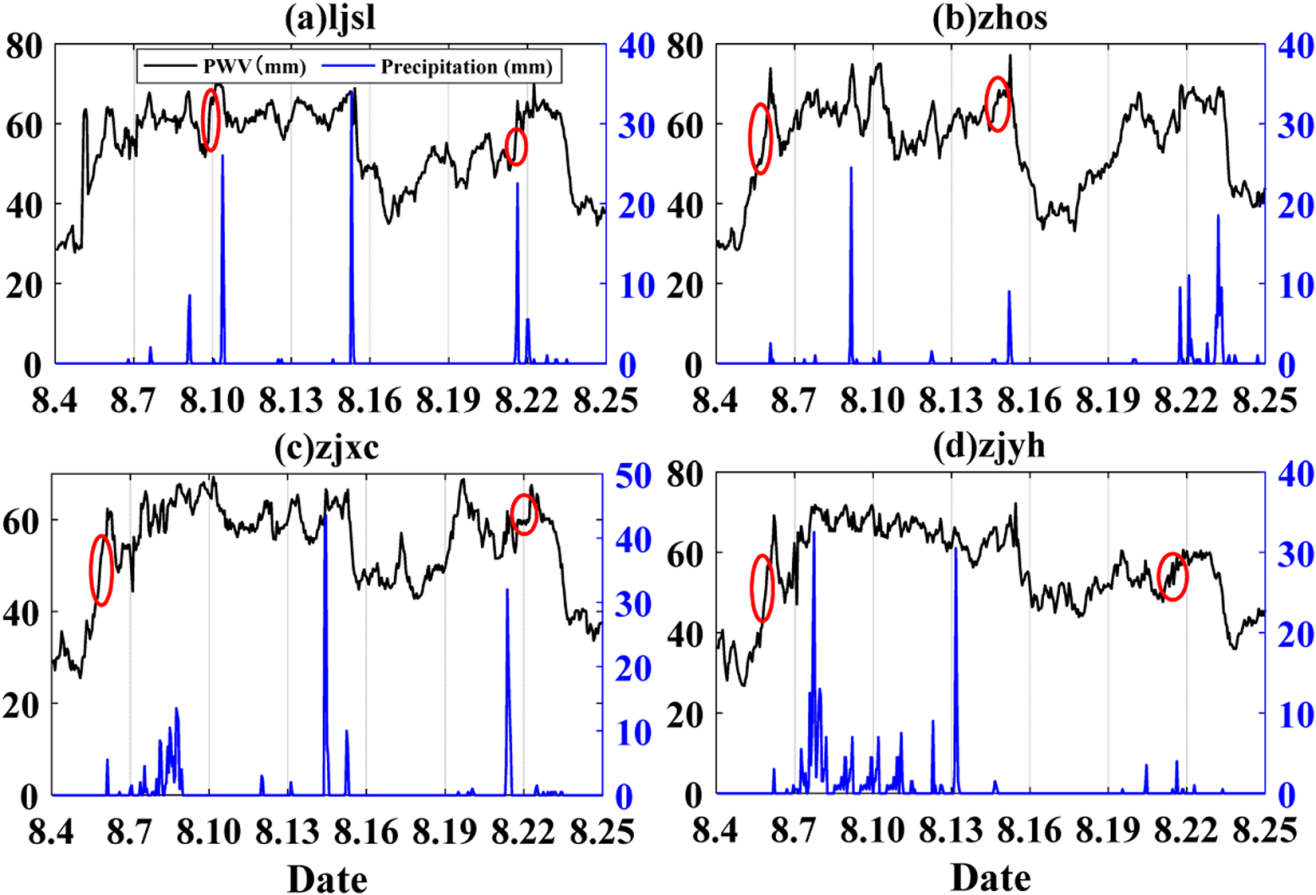
The relationship between PWV and rainfall from 4 August 2015 to 25 August 2015(four stations).Black line represents PWV value, blue line represents hourly rainfall, and red circle represents PWV variation before several rainfalls.

Hourly PWV data of four stations (LJSL, ZHOS, ZJXC, and ZJYH) and hourly rainfall information in the period of 4 August 2015 to 25 August 2015 are selected for further analysis. Figure 3 shows the relationship between PWV and rainfall in the selected period. In general, PWV increases before rainfall events because the occurrence of a rainfall requires enough water vapor supply. The rise of PWV occurs several hours before a rainfall, then as PWV decreases, rainfall begins to weaken and finally ceases.

To further explore the relationship between time-varying PWV and rainfall, the concept of PWV variation and the rate of change of PWV are defined as follows:

PWV variation = the maximum PWV before a rainfall−the minimum PWV before the adjacent maximum PWV

Interval epoch = time1 (corresponds to the maximum PWV)−time2 (corresponds to the minimum PWV)

The rate of change of PWV = PWV variation/interval epoch

The maximum and minimum of PWV, PWV variation and the rate of change of PWV are analyzed before several rainfall events (red circles, Fig. 3). The results are shown in Table 2.

**Table 2:** Statistical data of several rainfall events.

Rainfall event	Max PWV (mm)	Min PWV (mm)	ΔPWV (mm)	Interval (h)	The rate of change of PWV(mm/h)
LJSL(1)	71.1	64.9	6.2	6	1.03
LJSL(2)	51.1	48.3	2.8	3	0.93
ZHOS(1)	73.9	56.3	17.6	6	2.93
ZHOS(2)	55.8	51.3	4.5	3	1.50
ZJXC(1)	62.4	55.4	7	1	7.00
ZJXC(2)	67.5	58.9	8.6	5	1.72
ZJYH(1)	69.2	55.4	13.8	3	4.60
ZJYH(2)	57.3	51.5	5.8	3	1.93

Note: (1) and (2) are serial number of rainfall events shown by red circles in Fig. 3.

It can be seen from Table 2, before rainfalls, maximum PWV have reached high levels that all values are above 50 mm, and some even reach 70 mm. PWV variation are also large, and can reach 13.8 mm within 3 hours before a rainfall. And most of the rate of change of PWV are more than 1 mm/h. The results indicate that, as one of the necessary prerequisites for rainfalls, GNSS-derived PWV will rapidly increase and aggregate in a few hours before rainfalls.

From above analysis, it can be seen that GNSS-derived PWV and rainfall have a correlation relationship, which rainfalls occur in the rising period of PWV. This relationship can be used for short-term rainfalls forecasting.

### Analysis of the relationship between rainfall and single factors related to GNSS-PWV: PWV, PWV variation, and the rate of change of PWV, respectively

In this section, three factors -PWV, PWV variation, and the rate of change of PWV - are selected as rainfall forecasting factors. To verify the reliability of the three factors respectively, station LJSL is taken as an example for rainfall forecasting test (Tables 3 to 5, respectively).

**Table 3:** Forecasted results using monthly PWV thresholds.

Month	PWV threshold	Forecasted frequency	Correct rate (%)	False alarm rate (%)
2014.09	55.5	72/87	82.8	76.8
2014.10	44.8	2/2	100.0	92.3
2014.11	31.5	48/54	88.9	72.4
2014.12	23.0	16/19	84.2	77.5
2015.01	25.1	17/21	81.0	72.1
2015.02	27.0	48/56	85.7	56.8
2015.03	27.0	69/85	81.2	71.8
2015.04	31.5	20/24	83.3	91.9
2015.05	45.0	54/64	84.4	78.8
2015.06	58.5	67/81	82.7	81.7
2015.07	59.0	91/110	82.7	66.2
2015.08	55.0	29/36	80.6	90.4

Note: 72/87 represents 72 out of 87 events.

Due to the seasonal characteristics of PWV, the monthly average PWV varies widely, so the threshold of PWV is selected monthly. It can be seen from Table 3, at station LJSL, when only PWV threshold is used for forecasting, the forecasted correct rate is high, but it is accompanied by a high false alarm rate. And by changing thresholds, it can be found that the smaller the thresholds, the higher the correct rate, and also the higher the false alarm rate. Because when threshold is small, there will be contained more events which includes correct events and false events.

It can be seen from Tables 4 and 5 that, the larger the threshold, the greater the percentage of forecasted rainfall events in the threshold interval, which indicates that the rate of change of PWV and PWV variation are related to rainfalls forecasting. However, the larger the threshold, the less the forecasted frequency and the lower the correct rate. When the threshold of PWV variation is 1 mm, 87.79% of the rainfall events can be correctly forecasted. With the increase of the threshold, forecasted rainfall events and the correct rate decrease. When PWV variation is 5 mm, the correct rate decreases to 55.24%. Comparing Tables 4 and 5, the rate of change of PWV has a more sensitive response to short-term rainfalls forecasting than PWV variation, and it is time-independent. Above experiments and Benevides’s forecasted experiments in Portugal^21^ indicate that the rate of change of PWV can afford better forecasted results than other two factors (PWV, and PWV variation).

**Table 4:** Forecasted results using the thresholds of PWV variation.

PWV variation (mm)	Forecasted frequency	All rainfall events	Percentage (%)	Correct rate (%)
0	593	2782	21.32	92.80
1	561	2096	26.77	87.79
2	507	1591	31.87	79.34
3	449	1239	36.24	70.27
4	395	988	39.98	61.82
5	353	818	43.2	55.24

**Table 5:** Forecasted results using the thresholds of the rate of change of PWV.

The rate of change of PWV (mm/h)	Forecasted frequency	All rainfall events	Percentage (%)	Correct rate (%)
0	593	2782	21.32	92.80
0.2	580	2392	24.25	90.77
0.4	563	1862	30.24	88.11
0.6	487	1349	36.10	76.21
0.8	424	992	42.74	66.35
1.0	337	685	49.2	52.74

Note: The annual rainfall events at station LJSL is 639; ‘forecasted frequency’ refers to correctly forecasted events in the threshold interval; ‘all rainfall events’ refers to total forecasted event (including correctly forecast and falsely forecast) in the interval.

### Analysis of the relationship between the three-factor and rainfall

In the current researches, only one factor such as PWV or the rate of change of PWV is considered for short-term forecasting^17–21^, which the forecasted results are unsatisfactory. For example, the rate of change of PWV is used by Benevides for short-term rainfall forecasting, which the forecasted correct rate is about 75% and the false alarm rate is about 65%^21^. Therefore, in this section, three factors are all considered in rainfall forecasting to improve the forecasted results. Based on the above study, the rate of change of PWV is taken as a major factor, and monthly PWV and PWV variation are taken as auxiliary factors to reduce omission of rainfall events. The three-factor and the single factor (only the rate of change of PWV) are used to forecast short-term rainfalls and the forecasted correct rate are analyzed.

Table 6 shows the correct rate using the threshold of the rate of change of PWV. Table 7 shows the correct rate using the threshold of three-factor. It can be seen from the two tables that, with the increase of the rate of change of PWV, the forecasted rainfall events decrease. Comparing two tables found that, when the rate of change of PWV is the same, the results of the three-factor are better than the single factor. When the rate of change of PWV is 0.6 mm/h, for station LJSL, the correct rate using the single factor is 76.21% while the three-factor is 82.32%. The three factors can complement each other, which effectively reduce the omission of rainfall events and improve the correct rate. Compared with the single factor, using three-factor for short-term rainfall forecasting can achieve better results.

**Table 6:** Forecasted correct rate using the threshold of rate of change of PWV.

Station	The rate of change of PWV/(mm/h)				
	0.2	0.4	0.6	0.8	1.0
LJSL	90.77	88.11	76.21	66.35	52.74
ZHOS	91.19	89.31	80.03	71.86	59.91
ZJPH	88.37	82.78	74.32	60.73	46.83
ZJXC	90.57	85.71	79.47	68.65	54.79
ZJYH	91.73	84.07	71.60	59.01	46.30

**Table 7:** Forecasted correct rate using the threshold of three-factor.

Stations	The rate of change of PWV/(mm/h)				
	0.2	0.4	0.6	0.8	1.0
LJSL	90.92	88.58	82.32	76.06	68.23
ZHOS	91.67	88.94	83.65	80.35	78.14
ZJPH	88.67	83.38	77.79	68.58	61.48
ZJXC	91.12	87.10	81.55	75.87	70.60
ZJYH	91.85	85.80	80.49	77.41	75.43

Monthly PWV, PWV variation, and the rate of change of PWV can be used for short-term rainfall forecasting, in which the rate of change of PWV has the greatest effect. Therefore, a short-term rainfall forecasting method using three-factor is established in next section.

## Using GNSS-PWV for short-term rainfall forecasting

### The method establishment

In this section, three factors are used as predictors for short-term rainfall forecasting, and a forecasted method is proposed, in which the rate of change of PWV is the main factor, and monthly PWV and PWV variation are auxiliary factors, so that the forecasted results can be improved.

The method is established as follows:

1. Data processing, then PWV and rainfall data are obtained. According to different circumstances of stations, different sliding window are selected for data-fitting;
2. Polynomial fitting is used for each segment (7^th^ order polynomial is used here). To avoid accumulation of errors, the “translation” method is used to move each segment to zero, then the maximum and minimum of each segment is calculated, and the PWV variation and the rate of change of PWV are obtained;
3. Different thresholds are selected according to actual situation of stations. A forecasted window is selected (6 h is selected here), and successfully forecasted rainfalls and falsely forecasted rainfalls of the period are counted, then they are compared with actual rainfall events. Finally, the correct rate and the false alarm rate are calculated, and the forecasted results are received.

### Principle of threshold selection

Tables 6 and 7 show that forecasted results vary with thresholds. Therefore, threshold selection is a key part to the method. The purpose of threshold selection is to minimize the false alarm rate under the premise of a high correct rate.

The principle of threshold selection is as follow:

For the rate of change of PWV, as the main factor, it can be found that forecasted results of different stations are different when with the same threshold, which indicates that the threshold selection is related to location of stations. When there is a priori value of threshold in a location, it can be used as an empirical information to select the threshold for other events, which makes forecasted process easier. As auxiliary factors, for PWV, the threshold is selected monthly because of its seasonal characteristics, and should ensure that the correct rate is much higher than the false alarm rate; for PWV variations, it should be determined under the selection of the rate of change of PWV, to improve the forecasted results.

It can be seen from Table 7 that, the threshold is not unique, but a range. Taking station LJSL as an example, when the threshold is between 0.6 mm/h and 0.8 mm/h, the correct rate is high and the false alarm rate is low, which satisfied the selection principle. That is to say, the appropriate threshold can be selected from this range and not unique.

### Verification of the short-term rainfall forecasting method

In this section, the method is verified with above five stations.

**Figure 4:**
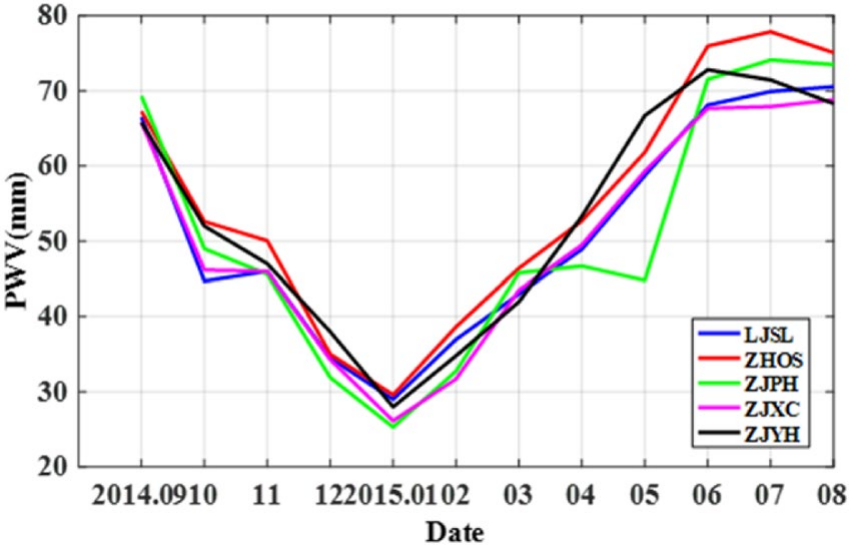
Monthly PWV threshold of five stations.

Figure 4 shows the selection of monthly PWV threshold. It can be seen that the threshold selection has a similar trend with the annual variation characteristic of PWV which is higher in summer and lower in winter and the differences among stations are small.

Table 8 shows the threshold of the rate of change of PWV and PWV variation, and forecasted results. When an appropriate threshold is selected, the correct rate is about 80% and the false alarm rate is about 66%. Compared the forecasted results with the previous studies (Benevides’s correct rate is about 75%^21^), the correct rate is improved with the value of about 7%, while the false alarm rate is similar (60% to 70%). In general, the method is feasible, applicable, and capable of high-accuracy short-term rainfall forecasting.

**Table 8:** Threshold selection and forecasted results of five stations.

Station	The rate of change of PWV (mm/h)	PWV variation (mm)	Correct rate (%)		False alarm rate (%)
			Rainfall	Large rainfall	
LJSL	0.6	6	82.32	95.38	65.21
ZHOS	0.8	3.5	80.35	92.11	66.11
ZJPH	0.6	3.5	77.79	86.49	68.35
ZJXC	0.6	3	81.55	91.67	65.27
ZJYH	0.6	1.8	80.49	86.76	66.18
Mean			80.50	90.48	66.22

Note: A large rainfall is regarded as a rainfall which hourly rainfall greater than 5 mm.

## Applications of the short-term rainfall forecasting method

To validate the method further, three actual rainfall events of different regions, different durations, and different types, are selected: the annual rainfall event in 2010 in Wuhan, the heavy rainfall event caused by Typhoon Phoenix in 2014 in Zhejiang, and a heavy rainfall event in June, 2015 in Zhejiang, respectively.

### The annual rainfall event in 2010 in Wuhan

Wuhan is located in the subtropical monsoon climate zone, and its precipitation is abundant. Over nearly 30 years, the average annual precipitation in Wuhan is 1269 mm, and rainfalls mainly concentrate from June to August. Except for seasonal variations, the fluctuations of annual PWV in Wuhan are small.

In this section, station WUHN is selected (longitude and latitude of 114.36° and 30.53°, respectively). The station is located in Wuhan University Information Department Observatory, and its adjacent rainfall station is JIED. The distance between two stations is less than 1 km. The selected time is full year 2010, and time resolution of the data is hourly.

**Figure 5:**
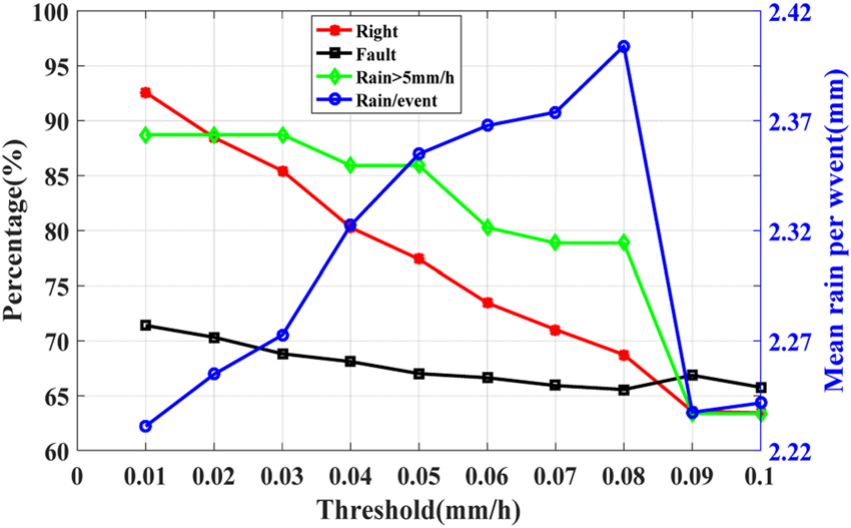
The forecasted results using the three-factor at station WHUN. The red polyline represents the correct rate, the green polyline represents the correct rate of heavy rainfall, the black polyline represents the false alarm rate, and the blue polyline represents the average rainfall (the total amount of forecasted precipitation divided by the number of forecasted rainfall events).

Figure 5 shows the effect of the three-factor on short-term rainfall forecasting. It can be seen that, when threshold increases, the correct rate decreases and the false alarm rate decreases. When the appropriate threshold is selected, the forecasted results can be received. For example, when the rate of change of PWV is selected as 0.04 mm/h, the correct rate is 80.3%, the false alarm rate is 68.1%, and the correct rate of heavy rainfall(Rainfall > 5 mm/h) is 85.9%. The forecasted results are consistent with the results of stations in Zhejiang, which indicates that the method is applicable, not only to the experimental area, but also to other regions.

### The heavy rainfall event caused by Typhoon Phoenix in 2014 in Zhejiang

Typhoon “Phoenix” landed in Ningbo, Xiangshan at 19:35, 22 September, 2014, and in its center, the max wind strength reached Category 10. On 23 September, Typhoon “Phoenix” crossed through Zhejiang Province and caused heavy rainfalls. Taizhou, Ningbo, Zhoushan, and other cities suffered small watershed flooding, landslides, and other disasters; some farmland were submerged, several traffic routes were cut, and water conservancy, electricity supply, and other infrastructures were damaged, which resulted in great loss.

Station XIAS and its adjacent rainfall station LIKA are selected; the time period from 20 September, 2014 to 23 September, 2014 (doy263 to doy266, four days in total) is selected. The typhoon landed Xiangshan in 22 September and rainfalls were concentrated on 21 and 22 September (doy264 and doy265). Because the rainfalls were heavy and happened in a short time, the forecasted window is changed to 20 minutes.

**Figure 6:**
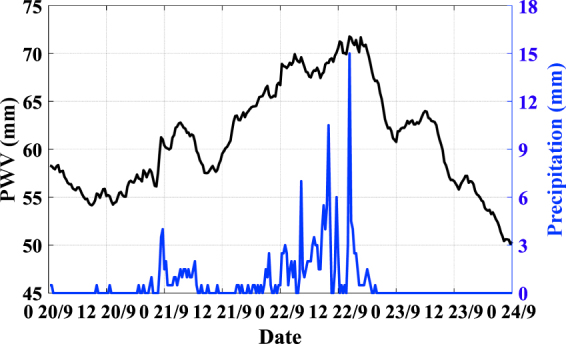
The relationship between PWV and rainfall of station XIAS at 20 to 23 September 2014. Black line represents PWV value, and blue line represents hourly accumulated rainfall.

Figure 6 shows the relationship between PWV and rainfall in selected period. It can be seen that PWV increases rapidly during rainfalls and the maximum rainfall approximately coincides with PWV peak.

**Figure 7:**
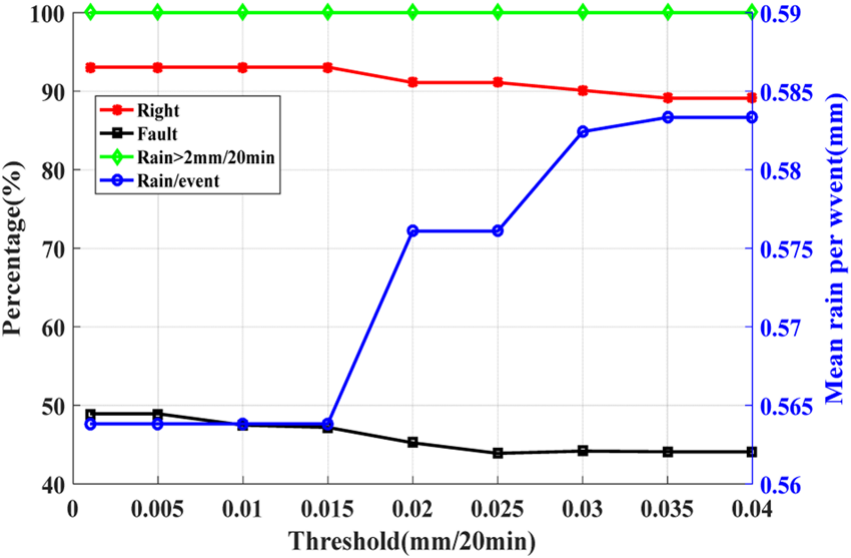
The forecasted results using the three-factor of station XIAS. The red polyline represents the correct rate, the green polyline represents the correct rate of heavy rainfall, the black polyline represents the false alarm rate, and the blue polyline represents the average rainfall (the total amount of forecasted precipitation divided by the number of forecasted rainfall events).

Figure 7 shows the effect of three-factor on rainfalls forecasting. It can be seen that, with the increase of threshold, the correct rate and the false alarm rate decrease slightly. When the rate of change of PWV is 0.025 mm/20 min, the correct rate reaches 91.1%, the false alarm rate is as low as 43.9%, and the correct rate of heavy rainfall (rainfall > 2 mm/20 min) is 100%. For different thresholds, the false alarm rate is always below 50%, and the correct rate of heavy rainfall is 100%, which indicates that the method can offer better prediction for heavy rainfalls.

### A heavy rainfall event in June, 2015, in Zhejiang

A heavy rainfall event occurred at 17, June, 2015 in Zhejiang Province. Due to high intensity and wide range of the rainfalls, five cities including Hangzhou, Taizhou, Quzhou, Jinhua, and Lishui, 28 counties, and 259 townships (a total population of 159.8 million) were affected; 2,278 houses collapsed; and direct economic losses reached 3.34 billion yuan.

Station ZJKH and its adjacent rainfall station KAIH are selected; the time period contained three days from 16 June, 2015 to 18 June, 2015 (doy167 to doy169). The rainfalls were concentrated on 17 June (doy168). Because the rainfalls were heavy and happened in a short time, the forecasted window was changed to 20 minutes.

**Figure 8:**
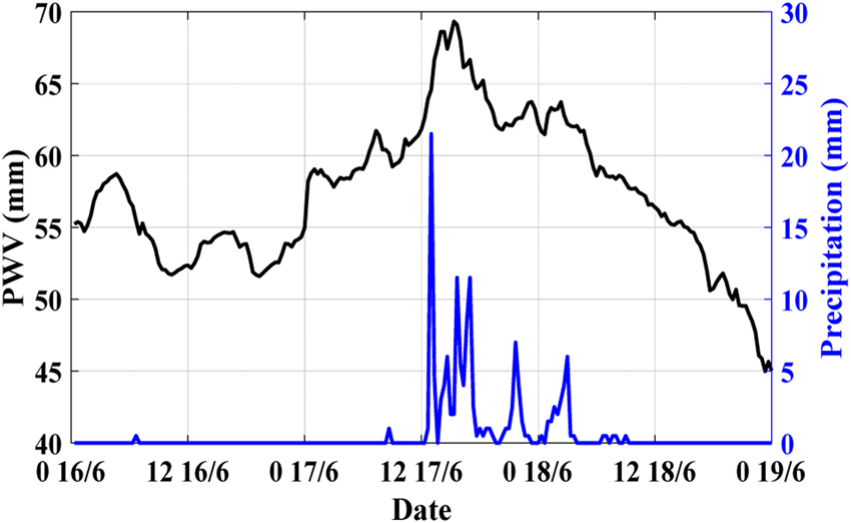
The relationship between PWV and rainfall of station ZJKH at 16 to 19 June 2015. Black line represents PWV value, and blue line represents hourly accumulated rainfall.

It can be seen from Fig. 8 that there is a correlation relationship between GNSS-derived PWV and rainfall. PWV continuously increased before rainfalls, when the conditions of rainfall were satisfied, rainfalls occurred. Then, when rainfalls stopped, PWV decreased, and maximum rainfall occurred near the PWV peak.

**Figure 9:**
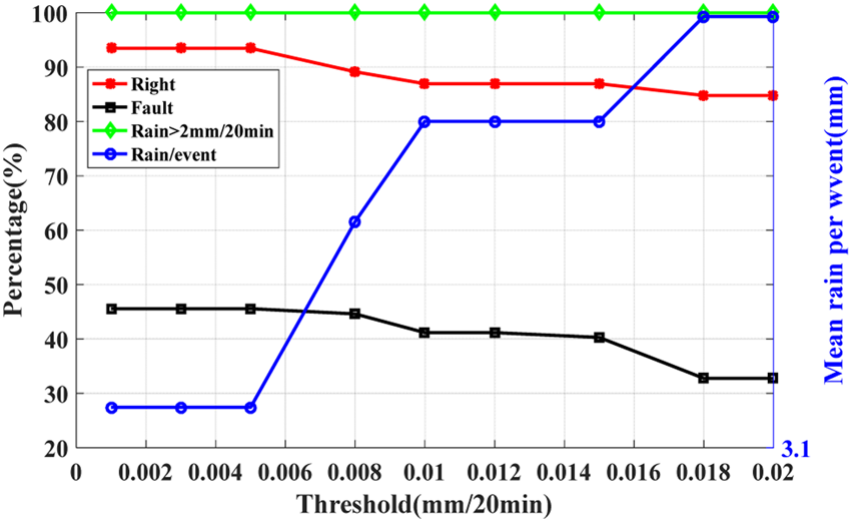
The forecasted results using the three-factor of station ZJKH. The red polyline represents the correct rate, the green polyline represents the correct rate of heavy rainfall the black polyline represents the false alarm rate, and the blue polyline represents the average rainfall (the total amount of forecasted precipitation divided by the number of forecasted rainfall events).

Figure 9 shows the effect of three-factor on rainfalls forecasting. It can be seen that the correct rate is above 85%, and the false alarm rate is below 50%. When the rate of change of PWV is 0.005 mm/20 min, the correct rate is 93.5%, the false alarm rate is as low as 45.6%, and the correct rate of heavy rainfall (rainfall > 2 mm/20 min) is 100%.

## Conclusions

Based on the relationship between GNSS-derived PWV and actual rainfalls, a short-term rainfall forecasting method is established. The method can forecast rainfalls by 6 hours in advance. It can detect about 80% of rainfall events. The method can be applied to different regions, different durations, and different types of rainfall events. The following conclusions can be drawn:

1. Most of China is located in monsoonal climate zone, so PWV shows seasonal characteristics which is higher in summer and lower in winter. There is a correlation relationship between PWV and rainfall, and maximum rainfalls usually occur near PWV peak.
2. A short-term rainfall forecasting method is established using the rate of change of PWV, PWV variation, and monthly PWV. The correct rate is about 80%, and the false alarm rate is about 65% and it shows high sensitivity to heavy rainfalls.
3. Compared to existing forecasted methods, monthly PWV and PWV variation are added as auxiliary factors, which improves the correct rate and reduces the false alarm rate. It can be seen from the forecasted results that the method can provide good predictions with different regions and types of rainfall events, thus it has wide applicability.
4. The method can be assimilated with other traditional weather forecasting techniques such as Numerical Weather Prediction (NWP) to improve the accuracy of short-term rainfall forecasting and warn the rainstorms beforehand timely.
